# Gas Chromatography-Mass Spectrometry Metabolites and Transcriptome Profiling Reveal Molecular Mechanisms and Differences in Terpene Biosynthesis in Two *Torrya grandis* Cultivars during Postharvest Ripening

**DOI:** 10.3390/ijms25115581

**Published:** 2024-05-21

**Authors:** Zuying Zhang, Lingling Gao, Liu Tao, Tianfang Wu, Jinwei Suo, Yuanyuan Hu, Weiyu Yu, Jiasheng Wu, Lili Song

**Affiliations:** State Key Laboratory of Subtropical Silviculture, Zhejiang A&F University, Hangzhou 311300, China; 451882@zafu.edu.cn (Z.Z.); gll@stu.zafu.edu.cn (L.G.); taoliu@stu.zafu.edu.cn (L.T.); wtf@stu.zafu.edu.cn (T.W.); sjw@zafu.edu.cn (J.S.); hyy_1985@zafu.edu.cn (Y.H.); yww888@zafu.edu.cn (W.Y.)

**Keywords:** *Torreya grandis*, GC-MS, terpene, transcriptome sequencing, gene expression

## Abstract

Terpene aroma compounds are key quality attributes of postharvest *Torreya grandis* nuts, contributing to their commercial value. However, terpene biosynthesis and regulatory networks in different *T. grandis* cvs. are still poorly understood. Here, chief cvs. ‘Xi Fei’ and ‘Xiangya Fei’ were investigated for their differences in terpene biosynthesis and gene expression levels during postharvest ripening using headspace solid-phase microextraction (HS-SPME) coupled with gas chromatography–mass spectrometry (GC-MS) and transcriptomic datasets. A total of 28 and 22 aroma compounds were identified in ‘Xi Fei’ and ‘Xiangya Fei’, respectively. Interestingly, differences in aroma composition between the two cvs. were mostly attributed to D-limonene and α-pinene levels as key determinants in *Torreya* nuts’ flavor. Further, transcriptome profiling, correlation analysis, and RT-qPCR annotated two novel genes, *TgTPS1* in ‘Xi Fei’ and *TgTPS2* in ‘Xiangya Fei’, involved in terpene biosynthesis. In addition, six transcription factors (TFs) with comparable expression patterns to *TgTPS1* and four TFs to *TgTPS2* were identified via correlation analysis of a volatile and transcriptome dataset to be involved in terpene biosynthesis. Our study provides novel insight into terpene biosynthesis and its regulation at the molecular level in *T. grandis* nut and presents a valuable reference for metabolic engineering and aroma improvement in this less explored nut.

## 1. Introduction

Nuts (walnuts, almonds, pistachios, etc.) are rich in protein and fat and provide a balanced blend of monounsaturated and polyunsaturated fatty acids, as well as a variety of bioactive compounds that play a beneficial role in disease management such as cancer, diabetes, and hypertension [1,2]. As a result, many studies have recommended the inclusion of nuts in a healthy diet [3]. In addition, the US Food and Drug Administration (FDA) approved the first specific health claim related to nuts in 2003—that eating 1.5 ounces (42.5 g) of nuts per day can reduce the risk of heart disease. Among nuts, *Torreya grandis* (*T. grandis*) is an important specialty nut from the subtropical mountains of China, with high nutritional value, and it contains a variety of bioactive compounds such as sciadonic acid, tocopherols, and flavonoids, which have preventive effects against heart and brain disease, as well as anti-inflammatory, antiviral, and antioxidant properties [4,5,6,7].

The aroma of the fruit is a complex mixture of hundreds of different heterogeneous volatile chemicals (terpenes, esters, alcohols, aldehydes, ketones, pyrazines, acids, etc.) in highly variable concentrations (ranging from a few mg kg^−1^ to a few ng kg^−1^) [8]. The different proportions of volatile components determine the overall aroma characteristics. The aroma also has a strong influence on fruit quality, with volatile components being the decisive factor between species [9,10]. At present, headspace solid-phase microextraction (HS-SPME) coupled with gas chromatography–mass spectrometry (GC-MS) is a widely used and efficient method for aroma detection and analysis [11,12,13,14,15,16]. Aroma means ‘Xiang’ and *T. grandis* means ‘Xiang Fei’ in Chinese. The fact that the Chinese name for *T. grandis* contains the word ‘Xiang’ implies that aroma is an important sensory quality of *T. grandis* nuts and that their characteristic aroma contributes significantly to their overall acceptance by consumers. Our previous study revealed five major categories of aromatic components in *T. grandis* nuts post harvest, including terpenes, aldehydes, alcohols, alkanes, and esters using SPME GC-MS analysis. Among them, terpenes constituted the major class throughout the postharvest ripening stage, ranging from 56% to 87% [17].

The terpene biosynthesis primarily occurs through two pathways: the mevalonic acid (MVA) pathway located in the cytosol and the 2-C-methyl-D-erythritol-4-phosphate (MEP) pathway located in the plastid [18]. The MVA pathway utilizes acetyl-CoA as a substrate to generate isopentenyl pyrophosphate (IPP) [19], which is mainly involved in the biosynthesis of sesquiterpenes, carotenoids, sterols, and triterpenoids in the cytosol [20]. The MEP pathway, on the other hand, utilizes pyruvate and glyceraldehyde-3-phosphate as substrates to produce dimethylallyl pyrophosphate (DMAPP) [21], primarily participating in the biosynthesis of monoterpenes, diterpenes, gibberellins, abscisic acid, and carotenoids in the plastid [22]. The primary enzymes that catalyze the manufacture of terpenes are called terpene synthases (TPSs). Since the discovery of the first linalool synthase gene in fairy fans (*Clarkia breweri*) [23], many *TPS* genes have been cloned and investigated in Arabidopsis (*Arabidopsis thaliana*) [24], citrus (*Citrus sinensis*) [25], grape (*Vitis vinifera*) [26], tomato (*Solanum lycopersicum*) [27], etc. Terpene biosynthesis is regulated though not only by key *TPSs* but also by the activity of transcription factors (TFs) [28]. Many TFs involved in terpene biosynthesis have been identified, such as AP2/ERF (APETALA2/ethylene-responsive factor), MYB, WRKY, bHLH (helix-loop-helix), bZIP (basic leucine zipper), and NAC (NAM, ATAF and CUC) families [29]. For example, CitAP2.10 promotes the production of the sesquiterpene (+)-valencene via activating the *CsTPS1* gene in ‘Newhall’ orange (*Citrus sinensis*) [30]. In peach fruit, PpbHLH1 is directly bound to the *PpTPS3* promoter to regulate linalool production [31]. Furthermore, SlMYC1, SlEOT1, and SlWRKY73 have been reported to activate *SlTPS5* promoter to promote linalool production in tomatoes [32].

Genotypes can also account for differences in terpene composition and content in different taxa represented by species, cv. type, etc. [33,34]. However, knowledge of the variation in terpene composition among different *T. grandis* cvs. during postharvest ripening is not reported in the literature. Therefore, this present study aimed to assess changes in terpene profiles from two *T. grandis* cvs. ‘Xi Fei’ and ‘Xiangya Fei’ during postharvest ripening as analyzed using headspace solid-phase microextraction (HS-SPME) coupled with the gas chromatography–mass spectrometry (GC-MS) technique. Furthermore, the regulatory mechanism underlying key gene expression patterns with terpene production in ‘Xi Fei’ and ‘Xiangya Fei’ was also investigated to underline the molecular mechanisms. The results presented from this study provide better insight into terpene biosynthesis and their regulatory mechanisms in *T. grandis* nuts.

## 2. Results and Discussion

### 2.1. Differences in Terpene Content Account for Aroma Differences between ‘Xi Fei’ and ‘Xiangya Fei’ Nuts during Postharvest Ripening

**Figure 1:**
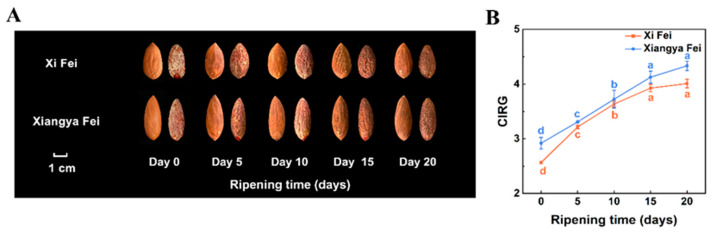
External changes in *T. grandis* ‘Xi Fei’ and ‘Xiangya Fei’ in different stages of postharvest ripening. (**A**) ‘Xi Fei’ and ‘Xiangya Fei’ in different stages of postharvest ripening treatment. (**B**) The CIRG value in the seed coat of ‘Xi Fei’ and ‘Xiangya Fei’. The different letters indicate significant differences (*p* < 0.05) in different stages of during postharvest ripening.

**Figure 2:**
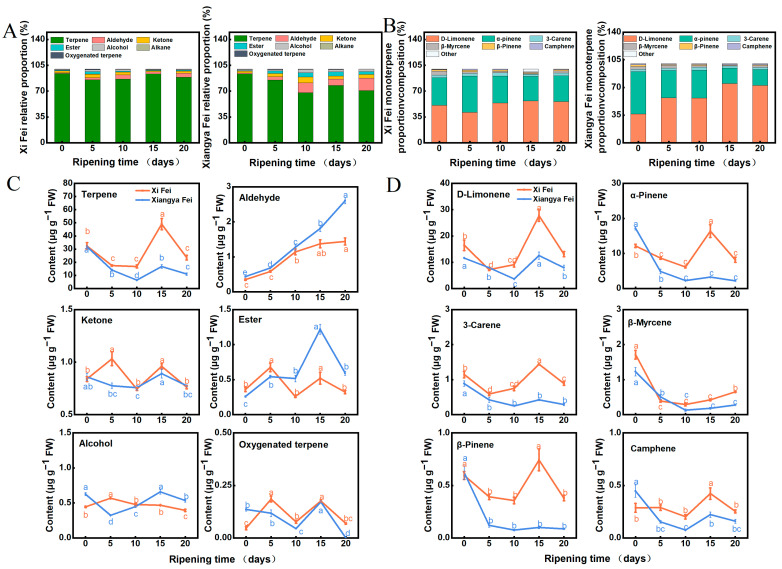
Changes in volatile composition in *T. grandis* ‘Xi Fei’ and ‘Xiangya Fei’ in different stages during postharvest ripening. (**A**) Changes in the proportion of total volatile substances in ‘Xi Fei’ and ‘Xiangya Fei’. (**B**) Changes in the proportion of total terpenes in ‘Xi Fei’ and ‘Xiangya Fei’. (**C**) Changes in the content of total volatile substances in ‘Xi Fei’ and ‘Xiangya Fei’. (**D**) Changes in the content of total terpenes in ‘Xi Fei’ and ‘Xiangya Fei’. Bars = 1 cm. Error bars represent SE based on three biological replicates. The different letters indicate significant differences (*p* < 0.05) in different stages during postharvest ripening. FW, fresh weight.

Two *T. grandis* cultivars, ‘Xi Fei’ and ‘Xiangya Fei’, were placed in a postharvest ripening environment at 25 °C and 90% relative humidity (RH), and then sampled on days 0, 5, 10, 15, and 20 (Figure 1A). Volatiles were analyzed using SPME coupled with GC-MS (Figure S1). Firstly, the CIRG value of seed coats of the two *T. grandis* cvs. during postharvest ripening was measured. The results revealed that the changes in CIRG value and seed coat color were consistent in both varieties, with a significant overall upward trend (*p* < 0.05; Figure 1B), revealing that the postharvest ripening process was proceeding normally [35]. Totals of 28 and 22 volatile compounds were identified from ‘Xi Fei’ and ‘Xiangya Fei’ using GC-MS, respectively (Tables S2 and S3). The results revealed that aroma compounds were mainly classified into seven major groups, including terpenes, alcohols, aldehydes, esters, oxygenated terpenes, alkanes, and ketones, with terpenes being the most abundant, accounting for 85.19–93.90% in ‘Xi Fei’ (Figure 2A) and 67.75–93.24% in ‘Xiangya Fei’ (Figure 2B), suggesting that terpenes are the main aroma-presenting substances during the postharvest ripening process of *T. grandis*. We previously explored changes in the aroma composition of ‘Xi Fei’ at 20 °C and a 90% RH environment during postharvest ripening, revealing that terpenes accounted for 56–87%, and that the terpene content increased significantly on days 4 and 8 [36]. Our current results showed that the total terpene content in both cvs. significantly peaked on days 10–15 of postharvest ripening, at 49.02 µg g^−1^ for ‘Xi Fei’ versus 16.70 µg g^−1^ for ‘Xiangya Fei’, with the terpene content of ‘Xi Fei’ being 2.94 times higher than that of ‘Xiangya Fei’ (*p* < 0.05; Figure 2C). Meanwhile, the content of other aroma compounds was lower and not significantly different compared to the total terpenes (Figure 2C). Different temperatures and humidity levels significantly influenced aroma content during postharvest ripening, in line with our previous study [17]. Upon further analysis of the terpenes, D-limonene, α-pinene, 3-carene, β-myrcene, β-pinene, and camphene were detected in the two cvs. to account for aroma, with D-limonene and α-pinene being the most abundant (Figure 2D). Differences in the levels of D-limonene and α-pinene were also observed among cvs., with D-limonene and α-pinene in ‘Xi Fei’ accounting for 41.06–56.61% and 33.35–49.48%, with 36.31–74.94% and 19.52–53.74% in ‘Xiangya Fei’, respectively (Figure 2D). Therefore, differences in the total terpene content are likely to account for the difference in aroma between these two cvs., and as observed for cv., impact on aroma composition in apple [37], bayberry [38], pepper [39], etc. In addition, the major difference in the two cvs. was D-limonene content (Figure 2D). D-limonene is a relatively abundant monoterpene that serves as the primary terpene element of most citrus fruit essential oils, which has a broad range of medicinal, antibacterial, antioxidant, and chemotherapeutic activities [40]. Moreover, D-limonene finds extensive application in the food and beverage, cosmetic, perfume, and pharmaceutical sectors [41]. *T. grandis* nuts have a lot of D-limonene, which may make it easier for it to be used extensively in industrial processing. Therefore, the major differences in terpene or D-limonene content in the two cvs. may provide a theoretical basis for variety selection and quality enhancement.

### 2.2. Deferentially Expressed Terpenoid Biosynthetic Genes Responsible for Terpene Accumulation in ‘Xi Fei’ and ‘Xiangya Fei’ during Post-Ripening Stage

**Figure 3:**
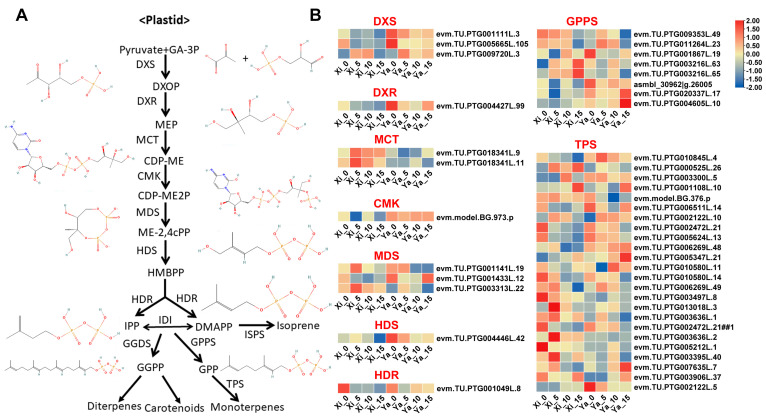
Analysis of MEP pathway genes of terpene biosynthesis in ‘Xi Fei’ and ‘Xiangya Fei’ nuts in different stages during postharvest ripening. (**A**) Plant terpene biosynthetic MEP pathway involved in monoterpene production [44]. Abbreviations: DXS, 1-deoxy-D-xylulose 5-phosphate synthase; DXR, 1-deoxy-D-xylulose 5-phosphate reductoisomerase; MCT, 2-C-methyl-D-erythritol 4-phosphate cytidylyltransferase; CMK, 4-(cytidine 50 -diphospho)-2-C-methyl-D-erythritol kinase; MDS, 2-C-methyl-D-erythritol 2,4- cyclodiphosphate synthase; HDS, (E)-4-hydroxy-3-methylbut-2-enyl diphosphate synthase; HDR, (E)-4-hydroxy-3-methylbut-2-enyl diphosphate reductase; GPPS, geranyl pyrophosphate synthase; GGPPS, geranylgeranyl pyrophosphate synthase; FPPS, farnesyl pyrophosphate synthase; TPS, terpene synthase; IPP, isopentenyl pyrophosphate; DMAPP, dimethylallyl pyrophosphate. (**B**) The FPKM value of MEP pathway genes in ‘Xi Fei’ and ‘Xiangya Fei’ in different stages of postharvest ripening treatment. The MEP pathway genes whose FPKM values were greater than 0 were selected based on annotation. ‘Xi’ represents ‘Xi Fei’, ‘Ya’ represents ‘Xiangya Fei’.

To investigate the molecular basis accounting for differences in terpene biosynthesis between these two *Torreya grandis* cultivars, transcriptome sequencing was performed on postharvest-treated samples of both cvs., resulting in 24 transcriptome samples. After filtering out low-quality reads, each sample yielded clean data of at least 8.74 G, with average Q20 and Q30 values of 97.25% and 92.75%, respectively, and an average GC content of 45.07% (Table S4). Additionally, principal component analysis (PCA) revealed clustering of the three biological replicates of ‘Xi Fei’ and ‘Xiangya Fei’ during the post-ripening stage, indicating that the transcriptomic data in our study were accurate for subsequent analysis (Figure S2). In this study, GC-MS analysis revealed that only for monoterpenes in *T. grandis* nuts did the aroma blend show differential levels among cvs. Therefore, the expression levels of 44 genes involved in monoterpene biosynthesis in the MEP pathway were analyzed (Figure 3). Several key regulatory genes in this pathway have been thoroughly studied, including DXS, which converts pyruvate and glyceraldehyde-3-phosphate to MEP and serves as a rate-limiting enzyme in the MEP pathway; DXR, the second rate-limiting enzyme in the MEP pathway that plays a crucial role in catalyzing terpenoid biosynthesis; GPPS, a short-chain isoprenyl transferase that provides the direct precursor geranyl diphosphate (GPP) for terpenoid biosynthesis [42]; and TPS, a terpene synthase that catalyzes the formation of sesquiterpenes, monoterpenes, diterpenes, and triterpene skeletons from different precursors [43].

**Figure 4:**
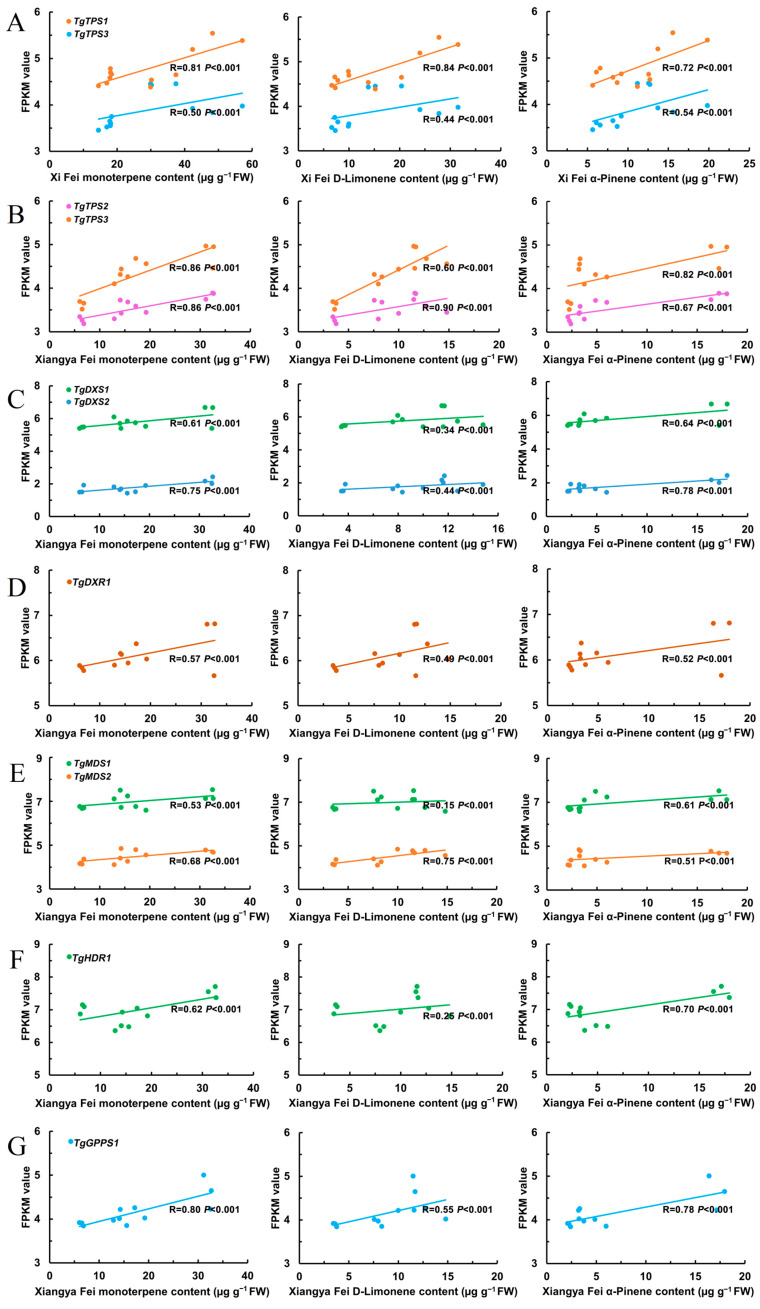
Correlation analysis of total terpene, D-limonene, and α-pinene contents and all related genes in MEP pathway in ‘Xi Fei’ and ‘Xiangya Fei’ in different stages during postharvest ripening. (**A**) The correlation analysis between *TgTPS1* and *TgTPS2* FPKM values and the total terpene, D-limonene, and α-pinene contents in ‘Xi Fei’. (**B**) The correlation analysis between *TgTPS2* and *TgTPS3* FPKM values and the total terpene, D-limonene, and α-pinene contents in ‘Xiangya Fei’. (**C**) The correlation analysis between *TgDXS1* and *TgDXS2* FPKM values and the total terpene, D-limonene, and α-pinene contents in ‘Xiangya Fei’. (**D**) The correlation analysis between *TgDXR1* FPKM values and the total terpene, D-limonene, and α-pinene contents in ‘Xiangya Fei’. (**E**) The correlation analysis between *TgMDS1* and *TgMDS2* FPKM values and the total terpene, D-limonene, and α-pinene contents in ‘Xiangya Fei’. (**F**) The correlation analysis between *TgHDR1* FPKM value and the total terpene, D-limonene, and α-pinene contents in ‘Xiangya Fei’. (**G**) The correlation analysis between *TgGPPS1* FPKM value and the total terpene, D-limonene, and α-pinene contents in ‘Xiangya Fei’.

**Figure 5:**
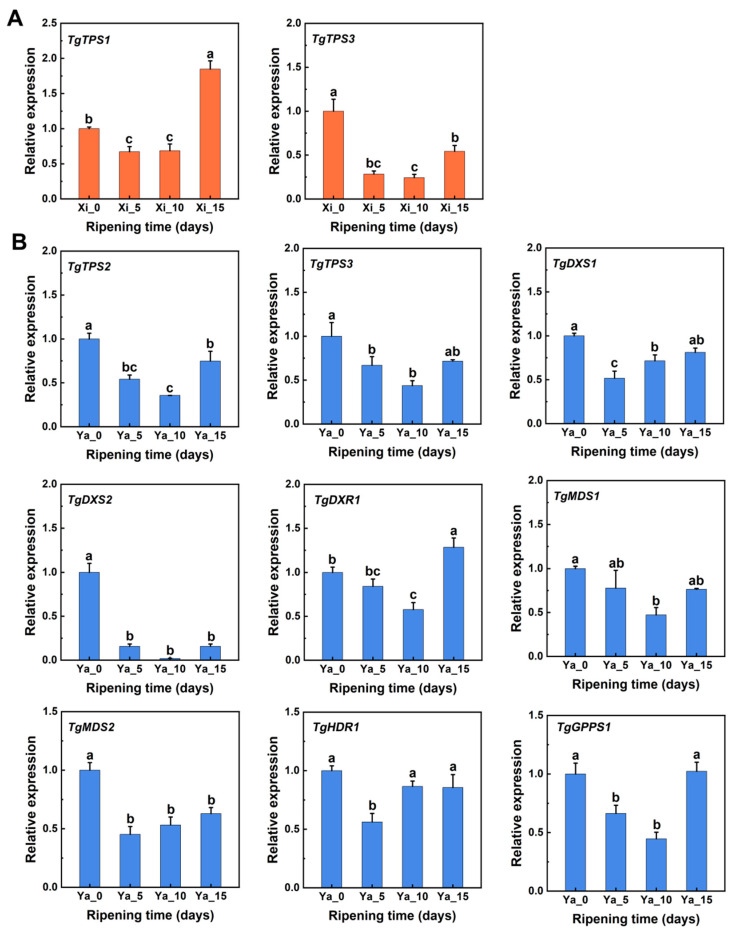
Relative gene expression of all related genes in MEP pathway in ‘Xi Fei’ (**A**) and ‘Xiangya Fei’ (**B**) cvs. in different stages during postharvest ripening. The data on Day 0 were set to 1. ‘Xi’ represents ‘Xi Fei’, ‘Ya’ represents ‘Xiangya Fei’. The different letters indicate significant differences (*p* < 0.05) in different stages of during postharvest ripening.

Some genes in the MEP pathway exhibited different expression patterns, and the differences in the expression levels of these genes may contribute to the total terpene differences among the two *T. grandis* cvs. To identify which specific genes or gene categories play a crucial role in terpene biosynthesis, a correlation analysis was conducted between the expression levels of the 44 genes in the MEP pathway and the total terpenes, D-limonene, and α-pinene in the two varieties. The results showed significant positive correlations between the FPKM value of certain genes and the content of total terpenes, viz., D-limonene, and α-pinene, including *TgTPS1* (evm.TU.PTG001108L.10) and *TgTPS3* (evm.TU.PTG006511L.14) in ‘Xi Fei’ (*p* < 0.05; Figure 4A). Among these, the FPKM value of *TgTPS1* (R = 0.81) in ‘Xi Fei’ showed a stronger correlation with the total terpene content than *TgTPS3* (R = 0.50) (*p* < 0.05; Figure 4A). In ‘Xiangya Fei’, nine MEP pathway genes were significantly correlated with the content of total terpenes, viz., D-limonene, and α-pinene, including *TgTPS2* (evm.model.BG.376.p), *TgTPS3* (evm.TU.PTG006511L.14) (*p* < 0.05; Figure 4B), *TgDXS1* (evm.TU.PTG001111L.3), *TgDXS2* (evm.TU.PTG005665L.105) (*p* < 0.05; Figure 4C), *TgDXR1* (evm.TU.PTG004427L.99) (*p* < 0.05; Figure 4D), *TgMDS1* (evm.TU.PTG001141L.19), *TgMDS2* (evm.TU.PTG001433L.12) (*p* < 0.05; Figure 4E), *TgHDR1* (evm.TU.PTG001049L.8) (*p* < 0.05; Figure 4F), and *TgGPPS1* (evm.TU.PTG001867L.19) (*p* < 0.05; Figure 4G). Meanwhile, expression results for these genes were further validated using RT-qPCR experiments (Figure 5). The RT-qPCR results were consistent with the FPKM value of the transcriptome data. Since genes such as *TgDXS*, *TgDXR*, *TgMDS*, *TgHDR*, and *TgGPPS* provided precursors for monoterpenoid biosynthesis and did not directly affect the content of total terpenes, this study mainly focused on the effects of *TPSs* on terpene biosynthesis. RT-PCR analysis revealed that *TgTPS1* in ‘Xi Fei’ and *TgTPS2* and *TgTPS3* ‘Xiangya Fei’ were their key terpene biosynthetic genes, and their expression differences accounted for differences in the total terpene content (Figure 5). Similar results were observed in two cultivars of *Dendrobium officinale* showing differentially expressed genes of 13 *TPSs* as the main factor accounting for differences in terpene composition [45].

### 2.3. Deferentially Expressed Transcription Factors Responsible for Variations in Terpenes in ‘Xi Fei’ and ‘Xiangya Fei’ during Postharvest Ripening

**Figure 6:**
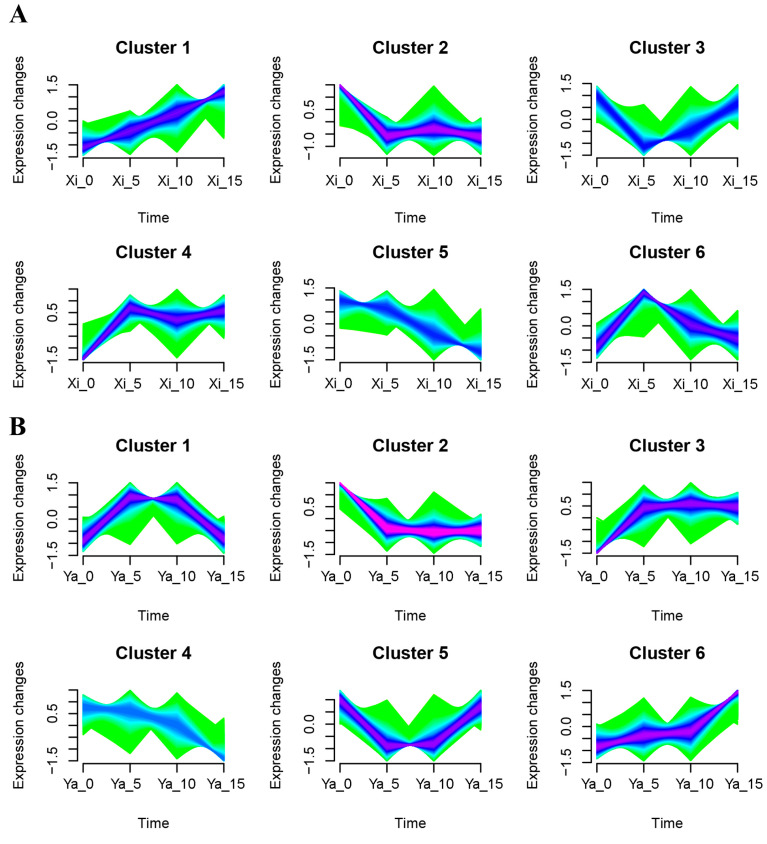
Mfuzz analysis of all genes from the transcriptomic datasets in ‘Xi Fei’ (**A**) and ‘Xiangya Fei’ (**B**) in different stages during postharvest ripening. ‘Xi’ represents ‘Xi Fei’, ‘Ya’ represents ‘Xiangya Fei’.

Identifying the molecular mechanism behind terpene biosynthesis is essential to improve terpene production through metabolic engineering. Transcription factors (TFs) play important roles in regulating gene expression in various plant biological processes including terpene production [46]. In this study, co-regulation cluster identification and analysis was performed based on the Mfuzz soft clustering method to reveal TFs with similar expression patterns to *TgTPS1*, *TgTPS2*, and *TgTPS3.* The results revealed that all genes in ‘Xi Fei’ could be classified into six clusters, with *TgTPS1* in Cluster 1, implying that the TFs co-expressed with *TgTPS1* could be selected from Cluster 1 (Figure 6A). In ‘Xiangya Fei’, *TgDXS1*, *TgDXS2*, *TgDXR1*, *TgHDR1*, *TgGPPS1*, and *TgTPS2* belonged to cluster 2, *TgMDS1* in cluster 4, and *TgMDS2* and *TgTPS3* in cluster 5 (Figure 6B). Since most of the genes that were significantly and positively correlated with terpene biosynthesis in ‘Xiangya Fei’ were located in cluster 2, TFs co-expressed with *TgTPS2* in cluster 2 were further analyzed in ‘Xiangya Fei’.

**Figure 7:**
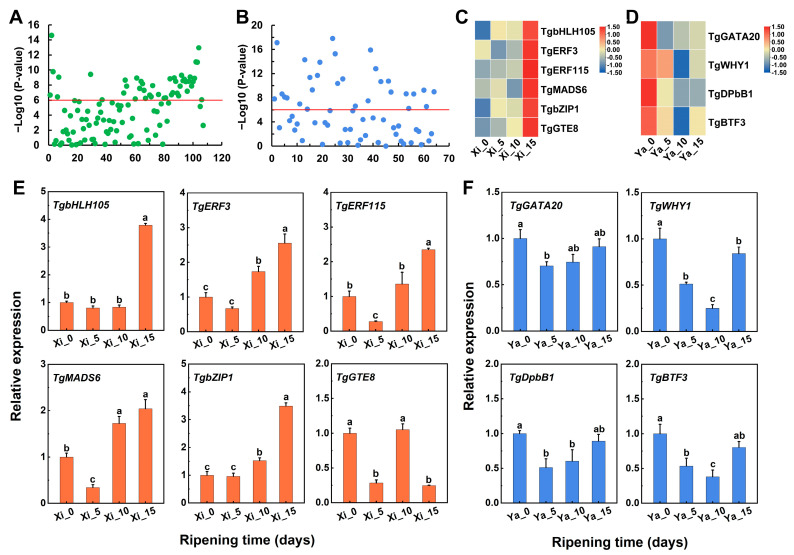
Statistical analysis of correlations between transcription factors (TFs) and genes related to terpene biosynthesis. (**A**) Statistical analysis of the correlation between candidate TFs and *TgTPS1* in ‘Xi Fei’. (**B**) Statistical analysis of the correlation between candidate TFs and *TgTPS2* in ‘Xiangya Fei’. (**C**) FPKM value of candidate TFs in ‘Xi Fei’. Threshold level −log_10_(*p*-value) > 6, R > 0.8. (**D**) FPKM value of candidate TFs in ‘Xiangya Fei’. Threshold level −log_10_(*p*-value) > 6, R > 0.6. (**E**) RT-qPCR analysis of candidate TFs in ‘Xi Fei’. (**F**) RT-qPCR analysis of candidate TFs in ‘Xiangya Fei’. The different letters indicate significant differences (*p* < 0.05) in different stages of during postharvest ripening.

A total of 107 TFs in Cluster 1 of ‘Xi Fei’ and 64 TFs in Cluster 2 of ‘Xiangya Fei’ were analyzed for correlation values with *TgTPS1* and *TgTPS2*, respectively (Figure 7A,B). Six TFs with −log_10_(*p*-value) > 6 and a correlation with *TgTPS1* > 0.8 were selected in ‘Xi Fei’, which were TgbHLH105 (evm.TU. PTG012091L.13), TgERF3 (evm.TU.PTG002487L.23), TgERF115 (evm.TU.PTG005873L.5), TgMADS6 (evm.TU.PTG006600L.3), TgbZIP1 (evm.TU. PTG004438L.39), and TgGTE8 (evm.TU.PTG000808L.2) (Figure 7C). In addition, four TFs with −log_10_(*p*-value) > 6 and a correlation with *TgTPS2* > 0.6 were selected in ‘Xiangya Fei’, namely TgGATA20 (evm.TU.PTG008279L.15), TgWHY1 (evm.TU.PTG022256L.4), TgTgDPbB1 (evm.TU.PTG005832L.37), and TgBTF3 (evm.TU.PTG001638L.23) (Figure 7D). To validate the expression patterns of TFs observed in correlation analysis, 10 annotated TFs were selected for analysis of their expression levels using RT-qPCR. The results revealed that the TFs involved in the regulation of terpene biosynthesis were different in ‘Xi Fei’ versus ‘Xiangya Fei’, and that the TFs were highly expressed in ‘Xi Fei’ on Day 15 of the post-ripening stage (Figure 7E). In contrast, TFs were highly expressed in ‘Xiangya Fei’ on Day 0 of the post-ripening stage (Figure 7F).

The main families of TFs involved in terpene biosynthesis in plants are bHLH [47,48], AP2/ERF [49], NAC [50], WRKY [51], MYB [52], and bZIP [53]. For example, terpene biosynthesis in *Litsea cubeba* was regulated by *LcbHLH78* and *LcTPS42*, and the overexpression of *LcbHLH78* significantly increased the content of α-pinene, linalool, and geraniol [54]. CitERF71 directly activates the *CitTPS16* promoter and promotes the synthesis of (E)-geraniol in sweet orange fruit [55]. However, there are fewer reports on the involvement of MADS, GTE, WHY, E2F/DP, and BTF3 transcription factor families in the regulation of terpene biosynthesis. Therefore, further study shall focus on clarifying the regulatory role of these TFs on *TPS* of terpene biosynthesis in *T. grandis* nuts.

## 3. Material and Methods

### 3.1. Plant Materials

The two primary *T. grandis* cvs. (‘Xi Fei’ and ‘Xiangya Fei’) with a distinct morphology difference were hand-picked on 10 September 2022 at the Anji *Torreya* base, Zhejiang Province, China (30°38′ N, 119°41′ E), put on ice, and transported to the lab within 5 h of harvest. The nuts were cracked (ca., 525 days after flowering) and the arils was removed mechanically. After washing with purified water, nuts that were free of mechanical damage and of uniform size and color were selected for subsequent experiments. The screened nuts were mixed, divided into three groups each of 5 kg, and then placed at 25 ± 2 °C and in a 90% RH ± 2% RH environment for postharvest ripening treatment [35]. Generally, the postharvest ripening process of *Torreya grandis* nuts usually takes a period of 10–15 days under appropriate temperature and humidity conditions [36]. Therefore, the nuts were then sampled on days 0, 5, 10, 15, and 20. After removing the seed shells, the kernels were cut into 1 mm × 1 mm pieces, snap-frozen in liquid nitrogen, and stored at −80 °C for subsequent experiments.

### 3.2. Determination of the Color of the Seed Coat

The color of the seed coat was determined using the color index of red grapes (CIRG) = (180 − H)/(L* + C) [56]. Every five days during the treatment, five to six *T. grandis* nuts from each of the two treatments were chosen at random and shelled to see how the seed coat color changed. A CR-10 colorimeter (KONICA MINOLTA, INC., Tokyo, Japan) was used to measure four spots at 90^◦^ intervals at the nut equator. The results showed that L* (lightness, 1~100 value range), a* (redness, −60~60 value range), and b* (yellowness, −60~60 value range) were recorded in the appropriate ranges. The hue’s intensity and purity were then measured by converting the a* and b* values into H (hue angle) [H = arc tan (b*/a*)] and C (chroma) [C = [(a*)^2^ + (b*)^2^]^0.5^].

### 3.3. Extraction of Volatile Compounds from T. grandis Nuts

Volatiles were extracted according to our previous study [36]. Volatile compounds from *T. grandis* nuts were extracted using a solid-phase microextraction (SPME) autosampler fitted with a 65 μm polydimethylsiloxane and divinylbenzene (PDMS-DVB) fiber (Supeclo Co., Bellefonte, PA, USA). The fiber was conditioned in a GC injector port at 250 °C for 30 min before use. Afterward, frozen *T. grandis* kernel tissue (1 g) was ground into a powder in liquid nitrogen and placed into 20 mL vials containing 5 mL of saturated NaCl solution. An internal standard, 2-octanol (0.76 mg/mL, 20 µL, GC ≥ 99.5%), was purchased from Sigma-Aldrich Co. (St. Louis, MO, USA) and then added and stirred for 10 s with a vortex before sealing the vials. The samples were extracted using the PDMS-DVB fiber with constant stirring after the samples were equilibrated at 40 °C for 30 min. Subsequently, volatiles were desorbed for 5 min at 230 °C into the splitless injection port of the GC–flame ionization detector (FID). For the volatile analysis, an Agilent 6890N GC with an FID and a DB-WAX column (0.25 mm, 30 m, 0.25 μm, J & W Scientific, Folsom, CA, USA) was employed. Triplicate analyses of each sample were performed to assess for variance.

### 3.4. Gas Chromatography-Mass Spectrometry (GC-MS) Analysis

The GC-MS analysis was performed based on our previous study [36]. Volatile compounds were subjected to GC analysis on an Agilent 6890A GC coupled to an Agilent 5975C MS and equipped with a DB-WAX column (30 m × 0.25 mm inner diameter, 0.25 μm phase film thickness, J & W Scientific, Folsom, CA, USA). The mass spectrometer was operated in electron impact mode at a voltage of 70 eV. The flow rate of helium through the DB-WAX column was 1.0 mL min^−1^. The injector temperature was 250 °C and the source temperature was 230 °C. The column was initially started at 40 °C; the temperature was then increased from 40 °C to 100 °C at 3 °C min^−1^ and finally increased to 245 °C at a rate of 5 °C min^−1^. The NIST Mass Spectral Library (NIST-08 and Flavor) was used to identify volatiles detected by GC-MS by comparing electron ionization mass spectra and retention time data. Volatile levels were determined relative to the peak area of the internal standard in each sample as a reference. To assess for sensitivity of the GC-MS method, an internal standard (2-octanol) curve spanning from 1.6 to 200 ug/mL was obtained and analyzed under the same conditions used for Torreya nut analysis. The calculated limit of detection (LOD) and limit of quantification (LOQ) were 0.484 and 1.472 ug/mL, respectively. The reproducibility of the analytical method was based on injections of three different concentrations (0.4, 0.8, and 8 ug/mL) of the internal standard 2-octanol following the same method on three different days in triplicates, revealing a low coefficient of variation ranging from 0.011 to 0.082.

### 3.5. RNA Extraction and cDNA Library Construction and Sequencing

The CATB method [57] was used to isolate *T. grandis* total nut RNA, which was then subjected to electrophoresis on a 1.2% agarose gel, and the absorbance was measured at 260 nm to evaluate the total RNA purity and quality. The cDNA libraries were constructed from a total of 3 µg RNA samples, according to the standard protocol. To guarantee that the obtained library had an effective concentration greater than 2 nM, it was measured using the Qubit2.0 Fluorometer (Thermo Fisher Scientific, Carlsbad, CA, USA) and Agilent 2100 Bioanalyzer (Agilent Technologies, Inc., Santa Clara, CA, USA). After the library was qualified, the different libraries were sequenced by an Illumina NovaSeq 6000 (Illumina, Inc., San Diego, CA, USA). After trimming reads containing adapters, reads containing N bases, and low-quality reads from the raw data, clean data (clean reads) were obtained. Based on the reference genome [58], the clean data were annotated accordingly.

### 3.6. Transcriptome Analysis

The gene expression levels were calculated as Fragments Per Kilobase per Million (FPKM). Differential expression analysis of the three conditions/groups (three biological replicates per condition) was performed using the DESeq2 R package (1.20.0). The resulting *p*-values were adjusted using Benjamini and Hochberg’s approach for controlling the false discovery rate. The threshold for significant differential expression was set as padj < 0.05 and |log2(foldchange)| > 1. The Kyoto Encyclopedia of Genes and Genomes (KEGG) was used to compare the pathway enrichment of the differentially expressed genes [4].

### 3.7. Real-Time qPCR (RT-qPCR) Analysis

RT-qPCR analysis was performed based on our previous study [36]. For cDNA synthesis, 1000 ng of total RNA from each sample was treated with a gDNA eraser to remove genomic DNA and was then used to synthesize first-strand cDNA using the PrimeScript™ RT Master Mix (Takara, Dalian, China). RT-qPCR was performed using a CFX96 instrument with the Ssofast Eva Green Supermix Kit (Bio-Rad Laboratories, Inc., Hercules, CA, USA). The primers for the RT-qPCR test were designed using NCBI/Primer-BLAST and the sequences of all primers are listed in Table S1. The qPCR reactions were performed in a total volume of 20 μL, consisting of 10 µL of SYBR PCR supermix (Bio-Rad Laboratories, Inc., Hercules, CA, USA), 6 µL of DEPC-treated H_2_O, 2 µL of 5-fold-diluted cDNA template, and 1 µL of each primer (10 µM). The *TgActin* gene was used as an internal control to normalize small differences in template amounts. The 2^(−ΔΔCt)^ method was used to calculate the expression levels of each gene, and the data on day 0 were set as 1. Three biological replicates were used for RT-qPCR analysis.

### 3.8. Co-Regulation Cluster Identification and Analysis

Mfuzz is a soft-clustering method based on the fuzzy c-means algorithm, which is capable of clustering genes based on similar expression profiles, where genes with the same expression trend may be involved in the same biological process [59]. All genes in ‘Xi Fei’ and ‘Xiangya Fei’ were clustered using Mfuzz analysis based on the transcriptome data. A total of 6 clusters were classified in both *T. grandis* cultivars accordingly.

### 3.9. Statistical Analysis

The experiments were conducted using a totally random design. The data are presented as mean ± standard deviation (SD). Significant differences in volatile compounds and gene expression levels in the two *T. grandis* cultivars obtained in the triplicate analysis were determined by one-way ANOVA in SPSS Statistics 20.0 (SPSS Inc., Chicago, IL, USA).

## 4. Conclusions

In this study, volatile profiles in two *T. grandis* cultivars ‘Xi Fei’ and ‘Xiangya Fei’ were investigated during postharvest ripening, revealing that terpenes were the most abundant types of volatile compounds contributing to nut aroma post harvest. In addition, the terpene level in ‘Xi Fei’ was 2.94 times higher than that of ‘Xiangya Fei’. The analysis of transcriptome sequencing showed 44 genes in the MEP pathway likely to be involved in terpene biosynthesis in ‘Xi Fei’ and ‘Xiangya Fei’. Combined correlation analysis and RT-qPCR indicated that *TgTPS1* in ‘Xi Fei’ and *TgTPS2* in ‘Xiangya Fei’ accounted for its terpene biosynthesis as well as differences in aroma composition. Furthermore, co-regulation cluster identification and RT-qPCR validation demonstrated that the transcription factors involved in terpene biosynthesis in the two cultivars were significantly different: TgbHLH105, TgERF3, TgERF115, TgMADS6, TgbZIP1, TgGTE8 in ‘Xi Fei’ and TgGATA20, TgWHY1, TgTgDPbB1, and TgBTF3 in ‘Xiangya Fei’. Collectively, the production of terpene in *T. grandis* nuts during postharvest ripening appears to be the result of a combination of complex metabolic pathways, including diverse physiological processes and molecular regulation mechanisms. Our study provides valuable clues to gain better insight into terpene production in *Torreya* nut, and a reference for metabolic engineering and aroma improvement in the future.

## Data Availability

The data presented in this study are available on request from the corresponding author.

## Supplementary Materials

The following supporting information can be downloaded at: https://www.mdpi.com/article/10.3390/ijms25115581/s1.
