# Supplementary material for: Gas Chromatography-Mass Spectrometry Metabolites and Transcriptome Profiling Reveal Molecular Mechanisms and Differences in Terpene Biosynthesis in Two Torrya grandis Cultivars during Postharvest Ripening
Source: Int J Mol Sci. 2024 May 21;25(11):5581. doi: 10.3390/ijms25115581 (PMC11171539; doi:10.3390/ijms25115581)
Supplement: Supplementary file 1 [file ijms-25-05581-s001.zip › ijms-2965861-supplementary.pdf]

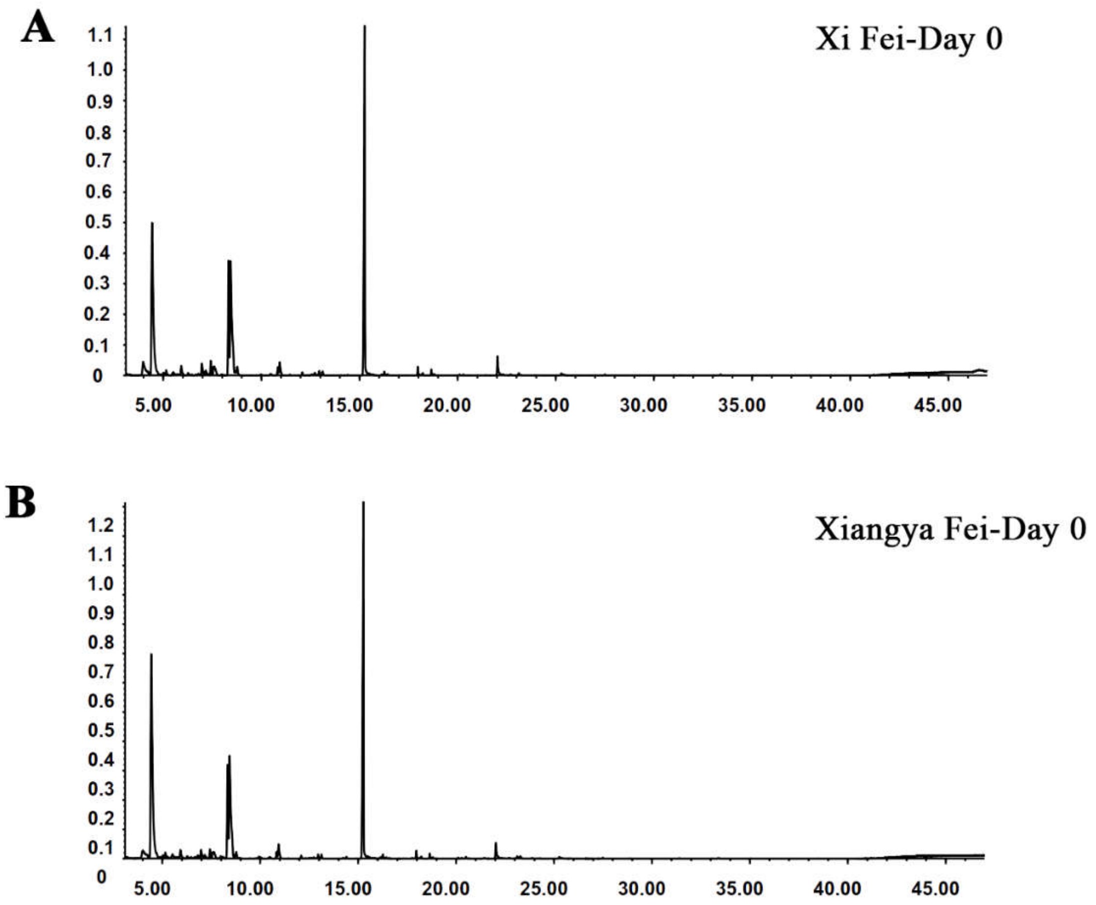

Figure S1. Gas chromatography-mass spectrometry (GC-MS) chromatogram of volatiles in *T. grandis* nuts determined. (A) 'Xi Fei' mass spectrogram on Day 0. (B) 'Xiangya Fei' mass spectrogram on Day 0.

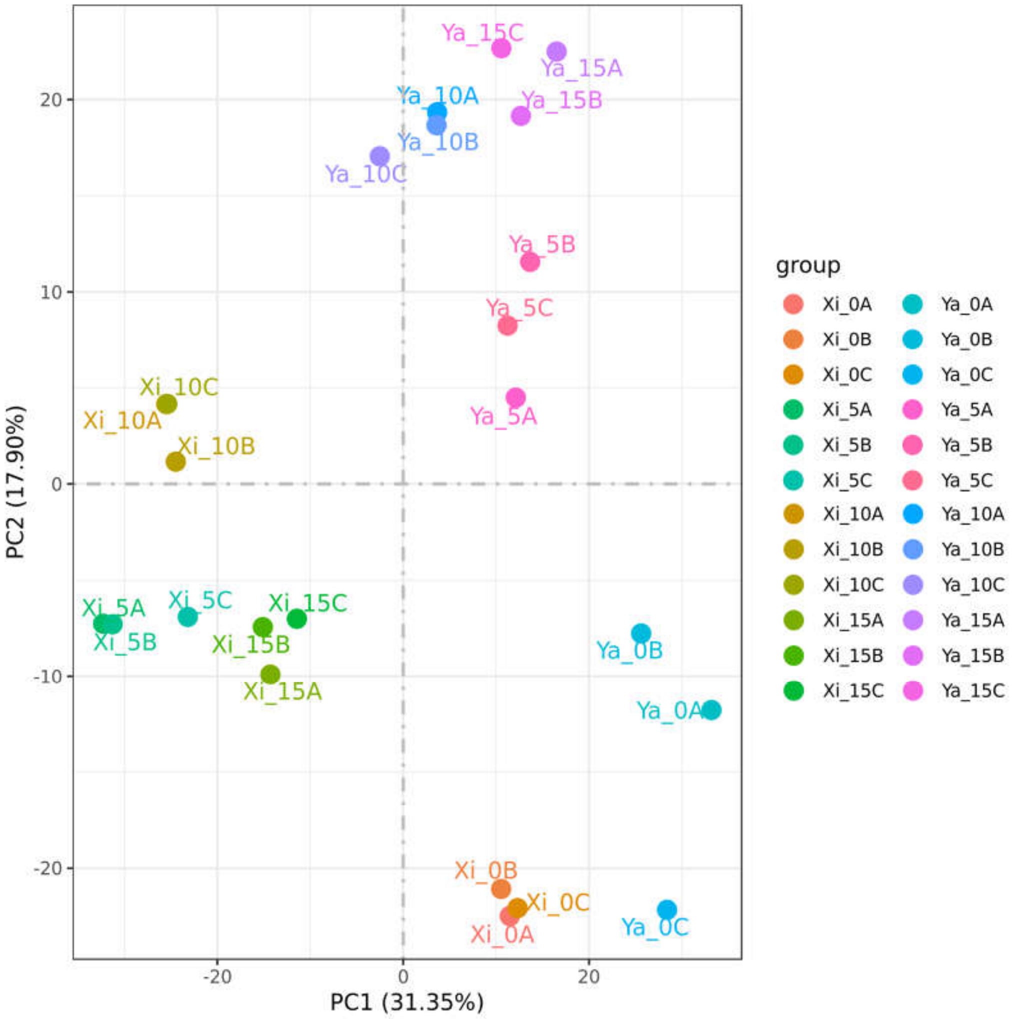

Figure S2. Principle component analysis of genes in *Torreya grandis* nuts at different ripening stages of Xi and Xiang. Xi_0, Xi_5, Xi_10, Xi_15 represented postharvest samples for 'Xi Fei' on Day 0, 5, 10, 15, respectively. Ya_0, Ya_5, Ya_10, Ya_15 represented postharvest samples for 'Xiangya Fei' on Day 0, 5, 10, 15, respectively.

Table S1. Primers used for reverse transcription (RT)-quantitative (q)PCR.

Gene	Forward (5'-3')	Reverse (5'-3')
TgActin	TGGCATCTCTCAGCACATTCCA	TGCCAACATCTCAAGCAAGCAC
TgTPS1	ATTTCATCTACTCTCCCCGC	GATTGAGAAGAACGTAGGGC
TgTPS2	CCTTTTCGAGCCAGTTACG	AACATATGGACGTGACCAAAG
TgTPS3	TTTGCTTCAATATAAACAGAGCG	GACTAGAAAGCCCACTTCAATA
TgDXS1	CATTGTTCATGAATGGAGGC	ATTTTGCTGACACAATCCCA
TgDXS2	CCATGATTTGGTTGGGATCTA	CGTATCCAGCAGAGGAGTTG
TgDXR1	CACATTTCTTCTTAGGAGAGACT	TGGAACCCAACTTTGAAGAC
TgMDS1	AGCAGAGTAGAAGCATGGC	CTCTTAATTGACTTGGATGGCT
TgMDS2	TTAAGTTTGTGAGACGACCAT	GGTGTTGGAGTTATTGCAGA
TgHDR1	CTCAGTGCCCAATACCCAA	CACAGTGCCGAATTTAACTGA
TgGPPS1	AATCGCAGCTTGTGAATGAA	AAGGCTCACACACTTGTCTA
TgbHLH105	TTTTGGCCCATCAATGACC	GCTTTCATTCCAGGTTTGCTA
TgERF3	GCATGTTCACACGTCAGT	AAGATGGAGGTAGGTTGAGAT
TgERF115	CAGATGCGTTTTCAACAGAC	ACTGACAATGCTGTGTAGGA
TgMADS6	GAACATTGCGAACTCCAGAA	TTTATGCATGCGACATCCTG
TgbZIP1	GGTTGTCTCAGGTTGGAAGA	GTACGCACAAAATTCAGAGG
TgGTE8	ATACCTGTTGGGAAATCGGA	GATCTCGCTTGACCCAATTC
TgGATA20	AAGAAGATGAAGAATCTTGGAG	ATGAGAAGTACAGCGCCTTC
TgWHY1	GCTGAATTTACAGTGATGCAGA	AAGTAACGCACATCTACTCG
TgDpbB1	CCAGTGAATGGATCTGCTAATAC	CAGGGTAGGAATCACAGATTTT
TgBTF3	AAGACTGACTCTGCTCCATA	ATGTCCGTTACTTGGGCT

Table S2. Main volatile compounds, and their retention time, molecular formula and aroma description in *T*orreya grandis nuts during postharvest ripening. All the volatile compounds can be divided into seven groups, including terpene, ester, aldehyde, alcohol, oxygenated terpene, alkane and ketone.

Peak No.	Retention time (min)	Compound	Formula	Class
1	3.527	Ethyl propionate	$C_5H_{10}O_2$	Ester
2	4.437	α-pinene	$C_{10}H_{16}$	Terpene
3	4.992	Ethyl 2-methylbutyrate	$C_7H_{14}O_2$	Ester
4	5.152	Camphene	$C_{10}H_{16}$	Terpene
5	5.513	Hexanal	$C_6H_{12}O$	Aldehyde
6	5.725	Undecane	$C_{11}H_{24}$	Alkane
7	5.908	β-Pinene	$C_{10}H_{16}$	Terpene
8	6.978	3-Carene	$C_{10}H_{16}$	Terpene
9	7.43	β-Myrcene	$C_{10}H_{16}$	Terpene
10	7.554	β-Phellandrene	$C_{10}H_{16}$	Terpene
11	7.928	α-Terpineol	$C_{10}H_{18}O$	Alcohol
12	8.317	D-Limonene	$C_{10}H_{16}$	Terpene
13	8.777	4-Ethylbenzoic acid, 2-ethylhexyl ester	$C_{17}H_{26}O_2$	Ester
14	9.21	Ethyl-β,β-dimethyl acrylate	$C_7H_{12}O_2$	Ester
15	9.753	γ-Terpinene	$C_{10}H_{16}$	Terpene
16	9.93	3-Methyl-3-buten-1-ol	$C_5H_{10}O$	Alcohol
17	9.959	(E)-Ocimene	$C_{10}H_{16}$	Terpene
18	10.022	1-Pentanethiol	$C_5H_{12}S$	Alcohol
19	10.834	Methyl benzoate	$C_8H_8O_2$	Ester
20	10.937	2-Octanone	$C_8H_{16}O$	Ketone
21	12.087	Isopentenyl alcohol	$C_5H_{10}O$	Alcohol
22	13.129	1-Hexanol	$C_6H_{14}O$	Alcohol
23	16.15	(*E*)-Limonene oxide	$C_{10}H_{16}O$	Oxide
24	16.265	1-Heptanol	$C_7H_{16}O$	Alcohol
25	17.306	Isooctyl alcohol	$C_8H_{18}O$	Alcohol
26	17.764	Camphor	$C_{10}H_{16}O$	Ketone
27	20.504	4-Terpineol	$C_{10}H_{18}O$	Alcohol
28	21.42	Benzeneacetaldehyde	$C_{10}H_{12}O$	Aldehyde

Table S3. The content of volatile components in different postharvest ripening stages of 'Xi Fei' and 'Xiangya Fei' by GC-MS (ng g^{-1}).

Compounds	Xi_0	Xi_5	Xi_10	Xi_15	Xi_20	Xiang_0	Xiang_5	Xiang_10	Xiang_15	Xiang_20
Ethyl propionate	0.00	49.13	31.92	0.00	0.00	0.00	0.00	0.00	0.00	0.00
α-pinene	12149.64	8668.10	6115.70	16349.42	8231.54	17164.27	4859.62	2246.42	3260.17	2197.43
Ethyl 2-methylbutyrate	0.00	357.46	0.00	0.00	0.00	0.00	0.00	0.00	0.00	0.00
Camphene	287.53	288.68	201.62	422.56	252.56	446.10	152.77	74.16	222.21	159.39
Hexanal	306.24	507.65	1032.58	1158.11	1277.34	390.42	582.39	1162.97	1597.19	2518.03
Undecane	69.47	0.00	0.00	0.00	0.00	0.00	0.00	0.00	0.00	0.00
β-Pinene	594.23	391.48	356.83	742.75	379.37	621.40	118.76	74.91	100.47	86.46
3-Carene	1142.94	587.19	748.17	1438.89	891.53	885.56	422.04	251.35	422.30	289.67
β-Myrcene	1712.50	389.82	291.55	421.14	648.01	1226.20	507.94	125.72	181.66	278.70
β-Phellandrene	0.00	0.00	0.00	1821.68	0.00	0.00	0.00	0.00	0.00	0.00
α-Terpineol	0.00	54.87	0.00	49.22	0.00	0.00	0.00	0.00	0.00	0.00
D-Limonene	16453.73	7191.67	9051.78	27750.81	13083.56	11595.67	7951.08	3603.16	12510.64	7931.51
4-Ethylbenzoic acid, 2-ethylhexyl ester	0.00	0.00	0.00	0.00	0.00	0.00	367.31	386.29	903.08	344.03
Ethyl-β,β-dimethyl acrylate	0.00	0.00	0.00	77.50	55.82	0.00	0.00	0.00	56.59	0.00
γ-Terpinene	0.00	0.00	0.00	77.09	0.00	0.00	0.00	0.00	0.00	0.00
3-Methyl-3-buten-1-ol	0.00	0.00	0.00	0.00	0.00	93.79	0.00	0.00	0.00	0.00
(E)-Ocimene	82.92	0.00	0.00	0.00	0.00	0.00	0.00	0.00	0.00	0.00
1-Pentanethiol	0.00	77.27	90.04	0.00	0.00	80.25	0.00	47.12	157.37	83.50
Methyl benzoate	358.89	268.61	227.33	443.01	264.62	258.43	176.60	130.06	258.96	247.38
2-Octanone	833.86	1010.89	732.49	932.52	760.69	835.55	732.07	742.11	871.11	775.45
Isopentenyl alcohol	129.11	48.99	49.92	47.20	21.64	107.15	18.33	33.70	67.86	30.97
1-Hexanol	155.82	248.20	161.51	232.17	186.94	181.62	131.66	192.21	257.85	239.04
(*E*)-Limonene oxide	46.89	101.80	76.86	99.91	68.55	65.55	49.87	44.60	112.10	0.00
1-Heptanol	159.36	173.58	177.17	187.91	185.67	162.84	172.03	174.37	174.42	180.22
Isooctyl alcohol	0.00	19.48	0.00	0.00	0.00	0.00	0.00	0.00	0.00	0.00
Camphor	4.84	21.36	10.93	26.95	0.00	23.83	43.61	14.00	20.74	0.00
4-Terpineol	0.00	28.28	0.00	26.76	0.00	69.88	67.52	0.00	59.31	0.00
Benzeneacetaldehyde	41.54	78.58	109.02	214.57	160.28	46.40	101.85	107.58	212.42	87.79

Table S4. Summary of sample data quality.

sample	library	raw_reads	raw_bases	clean_reads	clean_bases	error_rate	Q20	Q30	GC_pct
Xi_0A	FRAS22H000081-1r	68817704	10.32 G	66806386	10.02 G	0.03	97.98	94.04	45.19
Xi_0B	FRAS210269230-2r	67605314	10.14 G	62855908	9.43 G	0.03	96.77	91.7	45.36
Xi_0C	FRAS210269231-2r	60870732	9.13 G	60772920	9.12 G	0.03	96.76	91.7	45.38
Xi_5A	FRAS210269219-1r	60673646	9.1 G	60585772	9.09 G	0.03	97.05	92.36	45.38
Xi_5B	FRAS210269220-1r	70371454	10.56 G	63709610	9.56 G	0.03	96.92	92.16	45.78
Xi_5C	FRAS210240141-1r	71214392	10.68 G	66172546	9.93 G	0.03	97.76	93.97	45.63
Xi_10A	FRAS210240151-2r	68010152	10.2 G	63405346	9.51 G	0.02	97.99	94.37	45.29
Xi_10B	FRAS22H000061-2r	65521044	9.83 G	63984900	9.6 G	0.03	97.45	93.07	44.47
Xi_10C	FRAS210240151-1r	68671958	10.3 G	63614748	9.54 G	0.03	96.89	92.01	45.58
Xi_15A	FRAS22H000062-2r	64092506	9.61 G	62689552	9.4 G	0.03	97.58	93.32	44.81
Xi_15B	FRAS210269227-1r	70424282	10.56 G	65708632	9.86 G	0.03	97.73	93.92	45.95
Xi_15C	FRAS220003814-2r	72054518	10.81 G	66157666	9.92 G	0.03	97.07	92.4	45.08
Ya_0A	FRAS210269232-2r	63104112	9.47 G	58461220	8.77 G	0.03	96.71	91.52	44.94
Ya_0B	FRAS210269233-2r	77830324	11.67 G	72781046	10.92 G	0.02	98.21	94.79	44.95
Ya_0C	FRAS210269234-1r	71641982	10.75 G	66478940	9.97 G	0.02	98.09	94.37	44.3
Ya_5A	FRAS210240136-1r	64898520	9.73 G	58881832	8.83 G	0.03	96.76	91.77	45.33
Ya_5B	FRAS210240137-1r	71126794	10.67 G	64876664	9.73 G	0.03	96.95	92.18	45.04
Ya_5C	FRAS210240138-1r	67388244	10.11 G	62126372	9.32 G	0.03	97.09	92.41	45.08
Ya_10A	FRAS210269222-1r	63508394	9.53 G	58258168	8.74 G	0.03	96.73	91.72	44.9
Ya_10B	FRAS210240149-1r	68361416	10.25 G	64327780	9.65 G	0.03	97.61	93.6	44.68
Ya_10C	FRAS210269223-1r	67785252	10.17 G	61577062	9.24 G	0.03	96.72	91.7	45.01
Ya_15A	FRAS210240160-1r	64132640	9.62 G	59194730	8.88 G	0.03	96.91	92.01	44.53
Ya_15B	FRAS210240161-1r	68916364	10.34 G	63126024	9.47 G	0.03	97.24	92.68	44.48
Ya_15C	FRAS210269226-1r	69676158	10.45 G	63994366	9.6 G	0.03	97.06	92.33	44.6
